# TIGIT as a Promising Therapeutic Target in Autoimmune Diseases

**DOI:** 10.3389/fimmu.2022.911919

**Published:** 2022-06-03

**Authors:** Chenran Yue, Sheng Gao, Shuting Li, Zhouhang Xing, Hengrong Qian, Ying Hu, Wenqian Wang, Chunyan Hua

**Affiliations:** ^1^ School of Basic Medical Sciences, Wenzhou Medical University, Wenzhou, China; ^2^ Laboratory Animal Center, Wenzhou Medical University, Wenzhou, China; ^3^ School of the Second Clinical Medical Sciences, Wenzhou Medical University, Wenzhou, China; ^4^ Department of Surgery, The Second Affiliated Hospital and Yuying Children’s Hospital of Wenzhou Medical University, Wenzhou, China

**Keywords:** autoimmunity, TIGIT, immune regulation, therapeutic target, co-inhibitory receptors

## Abstract

Co-inhibitory receptors (IRs) are molecules that protect host against autoimmune reactions and maintain peripheral self-tolerance, playing an essential role in maintaining immune homeostasis. In view of the substantial clinical progresses of negative immune checkpoint blockade in cancer treatment, the role of IRs in autoimmune diseases is also obvious. Several advances highlighted the substantial impacts of T cell immunoglobulin and ITIM domain (TIGIT), a novel IR, in autoimmunity. Blockade of TIGIT pathway exacerbates multiple autoimmune diseases, whereas enhancement of TIGIT function has been shown to alleviate autoimmune settings in mice. These data suggested that TIGIT pathway can be manipulated to achieve durable tolerance to treat autoimmune disorders. In this review, we provide an overview of characteristics of TIGIT and its role in autoimmunity. We then discuss recent approaches and future directions to leverage our knowledge of TIGIT as therapeutic target in autoimmune diseases.

## 1 Introduction

Autoimmune diseases include a heterogeneous cluster of disorders affecting millions of individuals worldwide that are characterized by the imbalance of immunological tolerance and autoimmunity (1). Current treatment of autoimmune diseases is mainly based on systemic immunosuppression, which usually results in the risk of severe side effects (2). Therefore, a successful therapy is needed to reinstate long-lasting immune homeostasis without perturbation of normal immune function. Although the etiology and pathogenesis remain largely unknown (3), studies have demonstrated that dysfunction of co-inhibitory receptors (IRs) is involved in the development of autoimmune diseases (4, 5). IRs play an essential role in maintaining the balance between tolerance and autoimmunity, which have gained much attention as therapeutic targets for autoimmune disease settings.

T cell immunoglobulin and ITIM domain (TIGIT), a newly discovered IR, plays important roles in immune modulation. TIGIT together with CD226 forms a pathway that has striking similarities to the well-known CD28/CTLA-4 signaling pathway, with CD226 conducing positive signals, whereas TIGIT transmitting negative signals (6). The TIGIT axis has previously been reported to trigger immunological tolerance by suppressing autoreactive T cells, inducing tolerogenic dendritic cells (DCs), and promoting the generation and enhancing the suppressive capacity of regulatory T cells (Tregs) (7–9). Recently, evidence indicates that TIGIT is associated with the pathogenesis of multiple autoimmune diseases (10–15). Moreover, promising preclinical data using TIGIT-Ig fusion protein, agonist antibody for TIGIT or other approaches, show protective effects in murine models of autoimmune diseases (16–18). The scope of this review is to outline the characteristics of TIGIT, summarize its roles in multiple autoimmune diseases, and discuss the therapeutic potential and mechanism of TIGIT to regulate immune responses and to ameliorate disease activity in autoimmune disorders.

## 2 The Characteristics of TIGIT

### 2.1 Structure

TIGIT was first identified by Yu et al. in 2009 as an inhibitory receptor that mainly suppresses T cells activation (9). It is also known as WUCAM, Vstm3, and VSIG9, which belongs to the immunoglobulin (Ig) super family (19). This transmembrane glycoprotein consists of three domains, which are an extracellular Ig variable domain, a type I transmembrane domain and a short intracellular domain that possesses one immune-receptor tyrosine-based inhibitory motif (ITIM) and one immunoglobulin tyrosine tail (ITT)-like phosphorylation motif (20). There is 58% sequence homology between human TIGIT and murine TIGIT, and the ITIM-containing sequence of the cytoplasmic tail of TIGIT is the same in murine and human (9, 21). Similar to the inhibitory function of human TIGIT, mice TIGIT inhibits the cytotoxicity of mouse NK cells; Due to the cross-species specificity of the protein, the difference in the binding properties of human TIGIT and murine TIGIT which human TIGIT can bind more ligands to exert inhibitory effects; Whether the differences in the cross-species specificity of the human TIGIT and murine TIGIT will be further explored in the future (21).

### 2.2 Expression Pattern

TIGIT is predominately expressed on activated T cells and NK cells while TIGIT is not expressed on the initial CD45RA^+^CD4^+^ T cells. Similarly, compared with the expression levels of TIGIT on activated memory CD45RO^+^CD4^+^ T cells stimulated by CD3 and CD28 antibodies, the expression of TIGIT on resting memory CD45RO^+^CD4^+^ T cells is lower. However, this up-regulated expression level of TIGIT decreased rapidly after 6 days. In Tregs, TIGIT is mainly expressed on CD4^+^CD25^hi^ Tregs, and the expression increased after activation. It is also detected that the cells express TIGIT while also highly expressing Foxp3 and GITR (22, 23). A previous study demonstrated that TIGIT is expressed in all types of human NK cells to inhibit NK cytotoxicity by binding to PVR and PVRL through its ITIM region (24). TIGIT together with CD155, CD96, CD112, CD112R and CD226 belong to the members of PVR family (6).

Some transcriptional factors and epigenetic regulation mechanisms have been demonstrated to regulate the expression of TIGIT. A transcription factor Eomesodermin (Eomes) is expressed on CD8^+^ T cells of patients diagnosed with acute myeloid leukemia, and it can enhance the expression of TIGIT by binding to its promoter region (25). TIGIT can be expressed on follicular B helper T cells (Tfh) cells and be also present on some CD3^+^CD8^int^ T cells in tonsils (22). Tfh cells are characterized by low expression of the transcription factor Bach2, while overexpression of Bach2 in Tfh cells represses of a set of genes, especially TIGIT (26). Overexpression of a long non-coding RNA, maternally expressed gene 3 (MEG3) can up-regulate the expression of TIGIT in CD4^+^ T cells by absorbing miRNA-23a, and TIGIT^+^ Tregs inhibits the expansion of Th1 and Th17 cells to alleviate autoimmune-mediated aplastic anemia (27). Expression of TIGIT is also associated with mechanisms of methylation. For instance, hypomethylation and binding by Foxp3 at TIGIT locus contributes to the upregulated expression of TIGIT in Tregs (28).

### 2.3 Ligands

**Figure 1:**
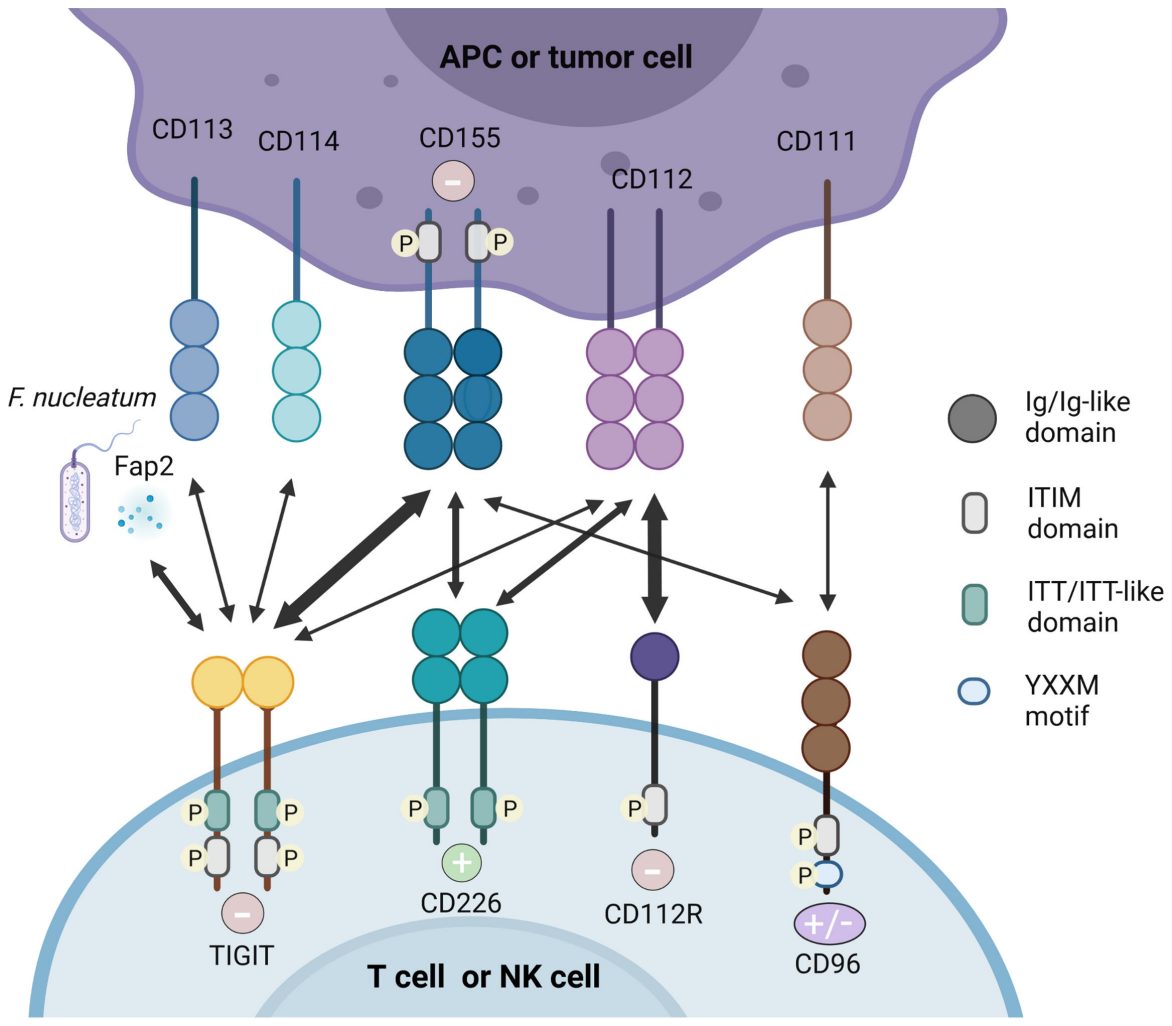
TIGIT/CD226 pathway axis. TIGIT, CD226, CD112R, and CD96 are expressed on activated T cells and NK cells. Their respective ligands, CD155, CD112, CD113, CD114, and CD111 are expressed on APCs or tumor cells. The Fap2 protein released by *F. nucleatum* is also identified as ligand for TIGIT. TIGIT, CD155, and CD112R which contain ITIM motifs in their cytoplasmic tail trigger inhibitory signals (–) to cells, while CD226 binds to CD155 and CD112 delivering an activating signal (+). CD96 contain ITIM, but human CD96 also contains an YXXM motif. Therefore, whether CD96 elicits a positive or negative signal in human T cells remain to be demonstrated. High affinity binding between receptors and their respective ligands are illustrated with heavy arrows, for example, the interaction between TIGIT and CD155.

Intriguingly, TIGIT has multiple ligands and it competes or shares with other inhibitory and stimulatory receptors for the same ligand (**Figure 1**).

#### 2.3.1 CD155

In both humans and mice, CD155 (PVR, necl-5) is identified as the physical ligand of TIGIT (29). As an Ig-like adhesion molecule, CD155 plays an important role in cell motility, natural killer and T cell-mediated immunity (30). CD155 is expressed on T cells, B cells, macrophages and DCs, and also weakly expressed in non-hematopoietic tissues such as the nervous system, kidney and intestine (22, 31). CD155 is frequently overexpressed in human malignant tumors (32, 33). As an immunomodulatory molecule, CD155 can combine with the costimulatory molecule CD226 and coinhibitory molecules TIGIT and CD96, therefore it plays a dual function in autoimmunity (30). Mouse CD96 can bind to CD111, but human CD96 cannot (34). In addition, human CD96 can also transmit stimulus signals, which is due to the Tyr-XX-Met box at its carboxyl end (where “X” is any amino acid), but mouse CD96 cannot (35).

Crystal structure analysis showed that TIGIT and CD155 both form homodimers, and form heterotetramers after ligand-receptor interaction (20). TIGIT has the highest affinity with CD155, while CD226 has the lowest affinity with CD155, which was demonstrated by direct radioligand binding assays and competition experiments (9). Therefore, TIGIT competes with CD226 to combine with greater affinity to CD155 indirectly and it can even bind to CD226 in *cis* and destroy the homodimer of CD226 to inhibit its signal transduction directly (19, 36, 37), showing its dominant inhibitory effect. Therefore, the balance between CD155/CD226 and CD155/TIGIT or CD155/CD96 plays an important role in maintaining normal NK and T cell functions (30).

The regulation of PVR gene *via* alternative splicing (AS) results in four AS isoforms. Two of them are transmembrane isoforms whereas the other two isoforms are soluble (37). Transmembrane PVRs were associated with activated CTLs after the demonstration of immune escape of hepatocellular carcinoma by reducing transmembrane PVRs (38). Conversely, high levels of soluble PVRs were observed in cancer patients which dampening the anti-tumor response mediated by CTLs (39). However, whether TIGIT bind to soluble PVRs needs further elucidation. Additionally, whether the competitive role between CD226 and TIGIT is correlated with diverse AS isoforms is still obscure (40).

#### 2.3.2 CD112

CD112 is also called PVRL2, NECTIN2 or PRR2 and was originally discovered as a PVR protein. CD112 is expressed in DCs, which is also widely expressed in hematopoietic and non-hematopoietic tissues such as pancreas, bone marrow, kidneys and lungs (38, 39). Compared with CD155, TIGIT has a weaker affinity with CD112 (9). By interacting with CD226, CD112 can stimulate the response of CTLs and NK cells (40). CD112 can also bind to CD112R, which preferentially expresses in T cells and inhibits the transduction of T cells related signals. Biacore experiments showed that CD112R has higher binding affinity to CD112 than CD226. Competitive experiment analysis indicated that CD226 can interfere with the binding of CD112 and CD112R, but TIGIT did not show this effect (41). However, the molecular and functional relationships between CD112R, CD226 and TIGIT need to be further explored.

#### 2.3.3 Other Ligands

TIGIT in mouse can also bind to CD113 (PVRL3), and the expression of CD113 is limited to non-hematopoietic tissues such as liver, testes, lungs, placenta and kidneys, but it has not been demonstrated to bind to human TIGIT (42). CD114 (Nectin4) has been recently found as a novel TIGIT ligand. Compared with other Nectins widely found in adult tissues (43), Nectin4 is abundant during fetal development, but its expression decreases in adulthood. However, in malignant tumors such as breast cancer, bladder cancer, lung cancer and pancreatic cancer, its expression has been returned (44). A recent study using fusion protein demonstrated the relationship between TIGIT and Nectin4, which is composed of the Fc part of human IgG1 and the extracellular part of a variety of tumor markers, including Nectin4, then stained NK cells to confirm that Nectin4 is a TIGIT ligand (45). By using micro thermophoresis (MST) experiment, they showed that both Nectin4 and PVR bind TIGIT with relatively high affinity. It confirmed that Nectin4 is a cancer-specific TIGIT ligand, and is the only member of the Nectin family that interacts with TIGIT alone. Moreover, the team has developed a monoclonal antibody against Nectin4 according to the characteristics of Nectin4 and demonstrated its efficacy *in vitro* and *in vivo*. The antibody represents the uniqueness between checkpoint inhibition and tumor specificity synergy, and its advantages may be proven in clinical use (45). The Fap2 protein expressed by *F. nucleatum* was identified as a ligand for TIGIT by using a library of *F. nucleatum* mutants (46).

### 2.4 Mechanisms of Action

The interaction of TIGIT expressed on NK cells and T cells with CD155 expressed on DCs forms a two-way signaling, which suppresses both the function of DCs and the activation of NK cells and T cells. It has been demonstrated that engagement of TIGIT by CD155 on human DCs or other antigen presenting cells (APCs) that expresses PVR inhibits IL-12p40 production and enhances IL-10 secretion by modulating the phosphorylation of p38 and Erk. *In vivo* administration of TIGIT-Fc also reduces the levels of inflammatory cytokines and induces the generation of tolerogenic DCs (9). In NK cells, TIGIT binds to CD155 and induces phosphorylation of tyrosine residues in the tail ITIM and ITT-like motifs through Src family kinases Fyn and Lck (4). It relies on the cytoplasmic adaptor growth factor receptor binding protein 2 (Grb2) and β-arrestin2 to recruit SH2-containing inositol phosphatase-1 (SHIP1) to the tail of TIGIT (47, 48). SHIP1 recruited to the tail of TIGIT by Grb2 inhibits phosphoinositide 3-kinase (PI3K) and mitogen-activated protein kinase (MAPK) signaling pathways, and leads to NK cell inhibition (47, 48). SHIP1 recruited by β-arrestin2 combines with ITT-like motifs and impairs TRAF6 self-ubiquitination and ultimately inhibits the activation of NF-κB, leading to the reduction of IFN-γ production in NK cells (4, 48). Similar to T cells, NK cells simultaneously express the co-stimulatory molecule CD226 and the co-suppressor molecule TIGIT, both of which bind to the same ligand, CD155. However, the TIGIT co-suppression signal is usually dominant under normal conditions because it has a higher affinity with CD155 than CD226 (21).

Engagement of TIGIT seems to block T cell priming, possibly *via* the TCR complex and promotes T cell survival by anti-apoptotic molecules (7). TIGIT signaling facilitates the inhibitory function of Tregs, because TIGIT^+^ Tregs express higher levels of CXCR3 and are more efficient in suppressing Th1 and Th17 responses (8, 36). Ligation of TIGIT by Fc-CD155 promotes the ability of Tregs to suppress Teff proliferation in specific conditions, reduces IFN-γ expression and corrects the suppressor defect of Tregs from patients with multiple sclerosis (18). The downstream of TIGIT signaling pathway in Tregs has also been explored recently. It has been demonstrated that TIGIT stimulation repressed PI3K while promoting suppression of Akt function which is accompanied by reduced phosphorylation of FoxO1 and nuclear localization (18). Previous results showed that activation of PI3K with phosphorylation of FoxO1 is required for Th1 reprogramming of Tregs (49). Therefore, this might be one mechanism whereby TIGIT suppresses the induction of Th1 programs. Inhibition of P13K by TIGIT resulted in suppression of mTOR (50). These findings further strengthen the connection between TIGIT signaling and PI3K signaling.

**Figure 2:**
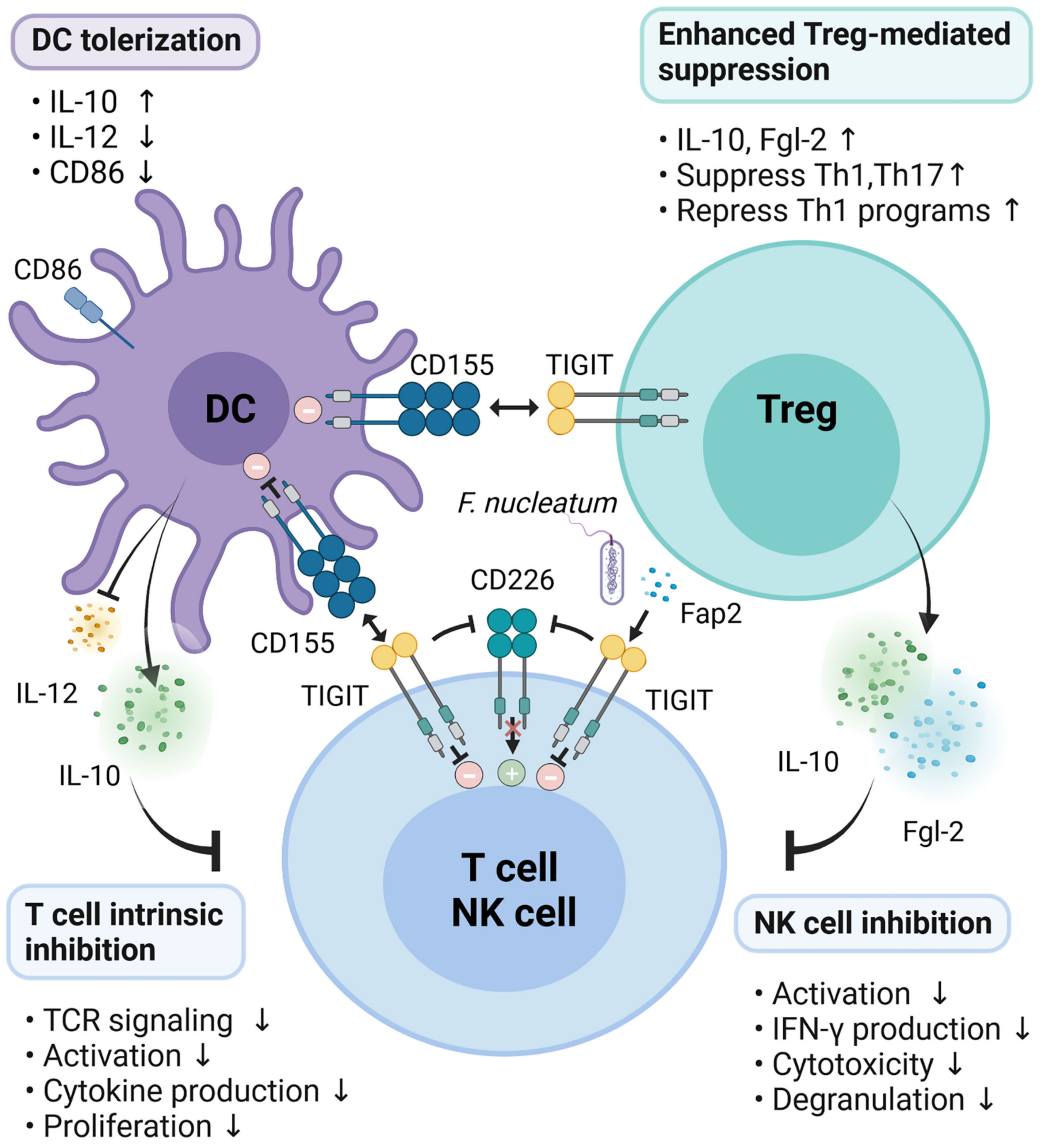
Mechanisms of TIGIT inhibition on immune responses. TIGIT expression on T/NK cells interacts with CD155 expressed on DCs or Fap2 protein form *F. nucleatum* to elicit direct inhibitory signals in T/NK cells. Engagement of CD155 on DCs by TIGIT induces immunosuppressive DCs by triggering IL-10 production while decreasing IL-12 secretion and CD86 expression, which indirectly inhibit T cells function. Activation of TIGIT enhances Treg-mediated suppression *via* secretion of IL-10 and Fgl2, suppression of Th1/Th17, and inhibition of Th1 programs. In addition, TIGIT disrupts CD226 homodimerization to impede CD226-mediated stimulatory signals.

The Fap2 protein of *F. nucleatum* inhibited the cytotoxic potential of NK cells and suppressed the activity of tumor-infiltrating T lymphocytes by interacting with TIGIT receptor to promote tumor survival (46). TIGIT suppresses T cell functions through several mechanisms to control immune response and maintain peripheral tolerance. Therefore, TIGIT enforcement is an attractive therapeutic to ameliorate autoimmune diseases (**Figure 2**).

## 3 Crucial Roles of TIGIT in Autoimmune Diseases

### 3.1 Rheumatoid Arthritis

Rheumatoid arthritis (RA) is a persistent inflammatory autoimmune disease, which leads to destruction of cartilage and bone in joint. The etiology and pathogenesis of RA are extremely complex and have not been fully elucidated so far, but factors such as genetics, environmental pressure, immune and cytokines disorders are involved in the occurrence of this disease (51, 52). CD4^+^ T cells are considered as the most important cells type that plays pivotal roles in the pathogenesis of RA by secreting IFN-γ and IL-17 (53). High levels of IFN-γ and IL-17 in synovial fluids and tissues are involved in the establishment of RA (54, 55), including the activation of immune cells and fibroblast-like synoviocytes (FLS), as well as bone and cartilage destruction.

Previous study has indicated that the frequency of TIGIT-positive CD4^+^ T cells in the synovial fluid (SF) of active RA patients was lower than that of inactive RA patients, and there is a negative correlation between disease activity and TIGIT expression (56). Overexpression of TIGIT by lentivector infection could hinder the function of CD4^+^ T cells, such as diminished IFN-γ and IL-17 production, and increased IL-10 expression. Moreover, up-regulated TIGIT ameliorated the severity of collagen-induced arthritis (CIA) model mice and reduced the production of anti-collagen II antibodies. These results suggested the potential therapeutic role of TIGIT in RA patients (56). CD226 has been established as the genetic risk factor for RA patients of gene sequencing analysis of blood cells and synovial tissue and it also participates in differentiation and function of Th17 cells (57, 58). Given the relationship between TIGIT and CD226, it is very important to regulate the roles of CD226 *via* TIGIT.

A central signature of RA is the progression into chronic and uncontrolled inflammation when without treatment. Exhausted CD4^+^ and CD8^+^ T cells develop into a hyperresponsive state and they are described in relation to inflammatory autoimmune diseases (59). T cells exhaustion is characterized by higher expression of co-inhibitory receptors, reduced production of cytokines, and limited proliferation (4). For example, upregulation of TIGIT is related to CD8^+^ T cells exhaustion (36, 60). CD8^+^ T cell exhaustion is associated with poor clearance of chronic viral infection, but conversely predicts better prognosis in autoimmune diseases (59). Greisen et al. reported that expression of TIGIT, PD-1, and TIM-3 was significantly increased on CD4^+^ T cells from RA synovial fluid mononuclear cells (SFMCs) compared to CD4^+^ T cells from both RA PBMCs and HC PBMCs and they found a positive correlation with TIGIT expression and disease activity (61). And this finding may demonstrate that the upregulation of these co-inhibitory molecules is correlated with CD4^+^ T cell exhaustion. In a prolonged inflammatory response like RA, exhausted T cells could develop into a hyporesponsive state with the progression of chronicity (62) while some of them could also be revived into functional T cells (63).

Although TIGIT has been associated with T cell exhaustion, it may have other roles as well. B cells activation and autoantibodies production are the pivotal events in the pathogenesis of RA, which lead to the formation of immune complex and tissue damage in joint. Tfh cells are well known to help germinal B cells differentiate into long lived plasma cells and produce high-titer antibodies (64). TIGIT is also expressed on Tfh cells and it was reported to act as a driver of immune response by some researches (65, 66).

The P2RX7 receptor is an ATP-gated cation channel. High concentrations of extracellular ATP can bind to the P2RX7 purinergic receptor, which ultimately leads to the maturation and release of the pro-inflammatory cytokines IL-1β and IL-18 through activating the NLRP3 inflammasome pathway. Therefore, blockade of P2RX7 has garnered much interest before as a potential strategy to treat autoimmune inflammatory diseases. However, it was recently discovered that P2RX7 has a negative effect on Peyer’s patch (PP) Tfh cells and promotes Tfh cell death while PARX7 deficiency worsen arthritis and promoted the production of auto-Abs (10). A recent study found that TIGIT expression is correlated with a reduction of Tfh cells apoptosis and this effect can be inhibited by P2RX7 receptor (10). Notably, the TIGIT expression increased significantly on P2rx7^-/-^ Tfh cells. It has been indicated that TIGIT engagement promotes T cell survival by up-regulating anti-apoptotic molecules such as Bcl-xL and receptors for pro-survival cytokines such as IL-2, IL-7, and IL-15 (7). Therefore, P2RX7 is the way to down-regulate TIGIT and inhibit the anti-apoptotic effect of TIGIT, which ultimately leads to cell death of Tfh population. Obviously, it is crucial to clarify the regulation of P2RX7 and TIGIT on apoptosis (10).

There is no doubt about the importance of Treg in autoimmune diseases owing to their critical role in maintaining immunological self-tolerance (67). In this regard, the reduction in the numbers and/or the impairment in the function and phenotypic defects of Tregs would lead to autoimmune intolerance and abnormal immune responses. Several studies demonstrated that levels of Tregs were significantly reduced in patients with RA (68, 69). Meanwhile, TIGIT expressed on Treg cells was considered to be involved in stability of Tregs and boost the inhibition functions of Tregs by impairing proinflammatory Th1 and Th17 cells (8, 70). However, recent data showed that the expression of TIGIT was elevated on Tregs in RA patients and there was no correlation between the expression of TIGIT and the disease activity of RA (27). And they also illustrated that TIGIT plays no significant roles in suppressive capacity of Tregs (27). This observation seems to be controversial in the light of inhibitory characteristics of TIGIT.

As was stated before, TIGIT is also expressed preferentially on NK cells and the activation of TIGIT axis can inhibit IFN-γ secretion and cytotoxicity in both human and mouse NK cells. NK cells expressed low levels of TIGIT possess a higher degranulation activity, cytokine secretion capability, and cytotoxic potential than that with high levels of TIGIT suggesting that TIGIT functions as a negative regulator of NK cells (71). In accordance with this, TIGIT expression on NK cells from patients with RA or SLE was significantly lower than that on NK cells from HCs (71). And its expression level was inversely correlated with the IFN-γ secretion capability of NK cells in patients with RA/SLE (71). Intriguingly, TIGIT expression levels show wide variation among individuals, which might explain one of the relevant mechanisms of the phenotypic and functional heterogeneity of NK cells and might be associated with susceptibility to autoimmune diseases (71). The above results indicate that the expression and function of TIGIT are different in distinct tissues, cell types and different stages of disease development. Therefore, the exquisite roles and mechanisms of TIGIT in the pathogenesis of RA merit further investigation.

### 3.2 Systemic Lupus Erythematosus

Systemic lupus erythematosus (SLE), a chronic inflammatory disease, is characterized by the loss of self-tolerance, the production of hallmark autoantibodies and the deposition of immune complexes, which ultimately lead to multiple organ damage (72). Serious complications especially the damage of renal function can further increase the morbidity and mortality of SLE (73). The etiology of SLE is complex, including genetic disorders, environmental factors, and changes in estrogen that trigger disruptions in innate and adaptive immunity (74). Current treatments of SLE patients mainly adopts empirical immunosuppressants to prevent disease progression, but their use increases the probability of infection in patients. Due to the complexity and heterogeneity of the disease, better therapeutic strategies that specifically target the pathogenic mechanism while maintain the homeostasis of the immune system are urgently required (75).

It is well known that during the occurrence and development of SLE, the activation of CD4^+^ T cells is crucial (76). Evidences from both clinical and basis experiments demonstrated that B cells hyperactivity and autoantibodies production are dependent on CD4^+^ T cells with abnormal costimulatory molecules (77, 78). Different from other inhibitory receptors, TIGIT is preferentially expressed on activated CD4^+^ T cells.

A significantly higher TIGIT expression on CD4**^+^** T lymphocytes was found in patients with SLE compared with HCs, especially in patients with higher levels of urine microalbumin, proteinuria, anti-Sm and anti-dsDNA (32, 79). C-reaction protein (CRP) levels and erythrocyte sedimentation rate (ESR) are also associated with the TIGIT expression on CD4**^+^** T lymphocytes in SLE (79). CD69, an important early activation marker could be used to evaluate the activation status of T cells in SLE. In addition, the frequency of CD69 on CD3**^+^** CD4^+^TIGIT^+^ T lymphocytes was higher compared to CD3**^+^** CD4^+^TIGIT^-^ T lymphocytes in SLE patients (32). Strangely, TIGIT expression on CD3^+^CD4^+^ T cells in patients of SLE was not associated with other clinical features, such as cutaneous manifestations, and oral ulcer (32). The data demonstrated that expression of TIGIT on CD4^+^ T cells is highly correlated with the SLE disease activity index (SLEDAI) suggesting TIGIT as a potential biomarker in monitoring disease activity in patients with SLE. The functional potential of TIGIT^+^CD4^+^ T cells including the proliferation, CD69 expression, and IFN-γ production was significantly lower than those of TIGIT^-^CD4^+^ T cells. In addition, TIGIT/CD155 engagement down-regulated the functions of CD4^+^ T cells from patients with SLE *in vitro* indicating TIGIT as a negative regulator of CD4^+^ T cell function in SLE and a potential therapeutic target for the treatment of this disease (79).

However, TIGIT expressed on different subsets of T lymphocytes including Tregs and Tfh cells shows different functions. Tregs which also express TIGIT play a key role in maintaining immune tolerance and preventing autoimmune responses by inhibiting the pro-inflammatory Th1 and Th17 cell responses. However, the number and function of Tregs in patients with SLE were impaired. It was reported that TIGIT-expressing Tfh cells exhibit strong functions of B-cell help cells (65). Regulatory follicular T (Tfr) cells display the dual characteristics of Tfh cells and Tregs. The expression of TIGIT are highest on Tfr cells within Treg population (80). Tfr cells which express high level of TIGIT repress the production of anti-dsDNA IgA in pristane-induced lupus mouse model (80). Nevertheless, a lower frequency of TIGIT on CD4^+^ T cells and CD8^+^ T cells was found in SLE patients with renal manifestations such as cylindruria, which suggests the crucial roles of TIGIT in homeostasis maintenance of relevant organs (11). Although the detailed functions of TIGIT in SLE need further investigation, the above data indicate that TIGIT, as an inhibitory costimulatory molecule, is associated with dysregulated activation of T cells in autoimmune responses.

Previous studies have confirmed the functional imbalance of NK cells in SLE patients. Consistent with its inhibitory characteristics, expression levels of TIGIT on NK cells are significantly lower in patients with RA/SLE than healthy individuals, and the decreased level of TIGIT on NK cells was more obvious in SLE patients compared to RA patients (71, 81). Furthermore, the frequency of TIGIT-expressing NK cells was significantly inversely correlated with the IFN-γ-producing capability of NK cells in both healthy individuals and in patients with RA or SLE (71). In an experiment on NK cells in the peripheral blood of SLE patients, TIGIT pathway blockade by functional anti-TIGIT monoclonal antibody was found to restore the secretion of IFN-γ by NK cells (81). In patients with active disease, the frequency of TIGIT-expressing NK cells was significantly lower than that in those with inactive disease indicating that TIGIT expression correlated negatively with disease activity and severity of SLE (81). These findings reveal that TIGIT exerts a forceful negative regulator effect on NK cells and TIGIT signaling pathway may be used as a potential therapeutic target for treating SLE.

Neutrophils as sentinels are important components of innate immunity to defense against pathogens. Previous evidence indicated that neutrophils also play a crucial role in the autoimmune responses and organ damage in the development of SLE (82). Several functional properties of lupus neutrophils were altered, such as aggregation increases, intravascular activation, diminished phagocytic capabilities and abnormal clearance of apoptotic substances (83). In 2016, Luo et al. firstly investigated the expression of costimulatory and coinhibitory molecules including PD-L1 and TIGIT on neutrophils in SLE. They reported that the frequency of PD-L1^+^ neutrophils but not TIGIT^+^ neutrophils was increased in patients with SLE when compared with healthy controls (84). In addition, PD-L1 expressing neutrophils were closely related to the disease activity and severity of SLE development, suggesting the potential of PD-L1^+^ neutrophils as negative feedback mechanism to prevent excessive autoimmune responses in the development of SLE (84). Although the frequency of TIGIT on neutrophils was no significantly changed, there might be other types of immune cells involved in process of SLE related with TIGIT. Therefore, the expression and roles of TIGIT on other types of immune cells in the condition of SLE require further exploration.

### 3.3 Inflammatory Bowel Diseases

Inflammatory bowel diseases (IBD) that include Crohn’s disease (CD) and ulcerative colitis (UC) are characterized by aberrant mucosal immune response triggered by genetic predisposition, gut microbes, and environmental risk factors (85, 86). Pro-inflammatory CD4^+^ effector T (Teff) cells migrate into and damage the intestinal organs by secreting inflammatory cytokines, which promote the development of IBD. On the contrary, Tregs can secrete anti-inflammatory cytokines to inhibit autoimmune inflammation, and promote tissue repair (87). An imbalance between Teff cells and Treg is crucial for the progression of IBD. Therefore, mechanisms underlying the modulation of this imbalance is required to maintain the gut homeostasis. Although the delicate mechanisms of IBD remains unknown while a growing body of evidence suggests that TIGIT is involved in the pathogenesis of IBD.

Surface levels of TIGIT were significantly lower on both inflamed mucosal CD4^+^ and CD8^+^ T cells of active IBD patients than that of the non-inflamed mucosa samples from remitting patients or the control samples (12). Therefore, levels of TIGIT might reflect the disease status of IBD. IL-15 is an important player in gut immune homeostasis. Treatment with IL-15 enhances TIGIT, but not CD226, on T cells (12). In IBD condition, the expression of TIGIT is more severely perturbed and is more prevalent than CD226 on mucosal T cells. In a recent study, Joosse et al. found that CD38^+^ Teff cells in peripheral blood of pediatric IBD patients with active disease contain lower population of cells expressing TIGIT. In addition, the majority of CD38^+^ Teff cells to express TIGIT but not FoxP3, and TIGIT instead of FoxP3 is positively correlated with IL-10 expression by CD4^+^ T cells (88). Therefore, the frequency of TIGIT^+^ cells in circulating CD38^+^ effector T cells can be used as an indicator to classify pediatric IBD patients and predict the severity of the disease course.

Tregs suppress inflammatory responses *via* immunoregulation or direct cytotoxic effects on Teff cells or APCs by granzymes and perforins (89, 90). Therefore, it is crucial to maintain immune homeostasis and could control excessive inflammation to ameliorate chronic colitis (91). TIGIT was demonstrated as a distinct marker for activated Tregs, and TIGIT expression on FoxP3^+^ Tregs was confirmed to enhance the suppressive effects of FoxP3^+^ Tregs (8, 56). In a dextran sulfate sodium (DSS)-induced chronic colitis mouse model, significantly lower frequencies of TIGIT^+^ Tregs were found in spleen, mesenteric lymph node (MLN), lamina propria mononuclear cells (LPMC), and colonic intraepithelial lymphocytes (IEL) compared to the control groups (92). Moreover, treatment with ERβ agonist ERB041 could significantly restore the frequency of TIGIT^+^ Tregs and alleviate DSS-induced chronic colitis and inflammation in mouse models, implicating the potential roles of TIGIT expressed on Tregs during the development of chronic colitis (92). Activation of TIGIT on FoxP3^+^ Tregs by CD155 on DCs lead to reduction of IL-12 production and it is closely associated with mucosal inflammation in UC. In active UC, the percentage of CD226^+^TIGIT^+^FoxP3^+^ Tregs were obviously increased. And the expression of both CD226 and TIGIT might be reliable biomarkers to evaluate the disease status of IBD patients (93).

NK cells are present in the intestinal mucosa under both the healthy and diseased conditions. Recently, a reduced number of peripheral NK cells was observed in UC patients (94). In UC, infiltration of immune cells into the intestinal mucosa could cause chronic inflammation. Some subtle but significant changes in immune cell frequencies and immune checkpoint expression were observed in patients with UC compared to HCs from a high-dimensional single-cell proteomics data by mass cytometry (94). The results also showed the increased expression of TIM-3 and TIGIT on NK cells, increased expression of CD155, PD-L1 and VISTA on monocytes, and decreased expression of TIGIT on CD4^+^ T cells (94). Strikingly, blocking TIGIT resulted in an increase of NK cell degranulation while blocking of CD226 got the opposite effects (94). Therefore, these studies provide the basis for further studies the TIGIT expression in IBD and demonstrate the possibility of targeted immune checkpoint to regulate immune cell effects in autoimmunity and chronic inflammation.

### 3.4 Type 1 Diabetes

Type 1 diabetes (T1D) is a sever chronic autoimmune disorder characterized by infiltration of autoreactive lymphoid cells into islets and impaired tolerance that promote destruction of insulin-producing β cells (95, 96). Immune tolerance of CD4^+^ T cells is very important in the prevention of autoimmune diseases. Tolerance of CD4^+^ T cells relies on thymus derived natural regulatory T cells (nTreg) which is programmed by transcription factor FOXP3. In a study of clinical samples of TD1, impaired differentiation or survival of nTreg lead to autoimmune destruction of pancreatic islet β cells. Moreover, the number of activated naïve nTreg and their signature genes FOXP3 and TIGIT can be modulated by the histone methyltransferase EZH2 which is a target of miRNA-26a. Increased expression of miRNA-26a was associated with decreased expression of EZH2 in pre-T1D (97).

Previous studies have demonstrated that blockade of IFN signal pathway delayed the onset of T1D however the underling mechanisms were unclear then (98, 99). Marro and coworkers reported that inhibition of IFN-α by using an antibody or a selective sphingosine-1-phosphate receptor 1 (S1PR1) agonist (CYM-5442) prevented T1D in mouse model and described the regulation mechanism. These treatments prevented the entry of autoimmune T cells into the islets, and thus protecting insulin-producing β cells from damage (100). CYM-5442 elicited the exhaustion signature in anti-self T cells showing as elevated expression of negative immune regulator genes including TIGIT, LAG3, and CTLA-4, etc. The enhanced expression of these molecules on autoreactive T cells limit the ability of autoimmune T-cell that might enter the islets from killing β cells (100). By these means, the production of insulin was preserved and glucose regulation maintained.

The therapeutic goal for T1D is to preserve β-cell function. Since T cells play crucial roles in the pathophysiology of T1D, much efforts aimed to find new therapies have been given to induce T cell unresponsiveness/tolerance (101). In newly diagnosed individuals with T1D, biologic therapies including anti-CD3 antibodies showed their effective roles. Teplizumab is an FcR-nonbinding anti-CD3 monoclonal antibody that achieved partial and transient preservation the function of β cells in clinical trials in onset T1D patients (102). T cell exhaustion is one of the mechanisms that cause T cell unresponsiveness *in vivo* and it acts as a beneficial prognostic indicator in autoimmune diseases (59, 103).

In a longitudinal study with clinical samples from T1D patients treated with teplizumab, Long et al. demonstrated that CD8^+^ T cells accumulated in the individuals with best response to teplizumab treatment and this population of CD8^+^ T cells exhibited high levels of multiple IRs such as TIGIT and KLRG1 (104). Nevertheless, the exhausted phenotype of these cells was not terminal because treatment with a recombinant ligand for TIGIT can further down-regulate the activation of these cells (104). These results suggest that regulating T cell exhaustion could be a potential intervention for T1D.

In the Autoimmunity-Blocking Antibody for Tolerance (AbATE) trial, subjects with onset T1D received two 14-day course of therapy with teplizumab. After two years, the preservation of plasma C-peptide, a surrogate for residual insulin-producing cells, was evaluated. In a follow-up study of this trial, the percentages of partially exhausted KLRG1^+^TIGIT^+^ CD8 T cells were induced within two months of teplizumab therapy and they can persist for about nine months after each course (105). The combination of markers induced by teplizumab on CD8 T cells in the above studies strongly suggest that TIGIT axis may be an important indicator of clinical outcome in T1D.

Several studies linked TIGIT expressing Tregs with diabetes in mouse models. TIGIT^+^ Tregs have been identified in the islets of NOD mice (106). In TCR-transgenic or retrogenic (Rg) mice model, deletion of Tregs leads to accelerated diabetes (107, 108). Development of autoantigen-specific vaccination is urgently needed to prevent islet autoimmunity. T1D mouse models showed that insulin acts as an essential autoantigen, which highlights the effects of insulin in initiating T1D autoimmunity. Serr et al. provides evidence that subimmunogenic vaccination with strong agonistic insulin mimotopes promoted human Foxp3^+^ Treg induction in human haematopoietic stem cell-engrafted NSG-HLA-DQ8 transgenic mice and prevented the onset of T1D *in vivo* (109). In children at risk of T1D, the T1D vaccine candidates could induce autoantigen specific Tregs for prevention of islet autoimmunity. Such induced human Tregs from humanized mic are stable, and harbour increased expression of Treg signature genes such as Foxp3, CTLA-4, IL-2Ra and TIGIT (109). Sprouse et al. used a two-TCR model to investigate the roles of TCR affinity in Tregs function during autoimmune diabetes. Results showed that Tregs with high- and low-affinity were recruited to pancreas participate in the protection from autoimmunity (110). It was observed that expression of TIGIT and IL-10 was significantly higher in high-affinity Tregs, whereas the enhanced transcripts for Areg and Ebits were displayed in low-affinity cells. The data suggest the distinct roles for high- and low-affinity Tregs in controlling autoimmunity (110). Another study revealed that NK cells from the subjects with T1D exhibited elevated level of CD226, and a higher CD226:TIGIT ratio as compared to HCs (13). The combination of IL-12 and IL-18 synergistically increased the expression of both costimulatory/co-inhibitory receptors CD226 and TIGIT, which enhance NK cell cytotoxicity that disrupt immunoregulation by Tregs in the disease (13).

### 3.5 Multiple Sclerosis

Multiple sclerosis (MS) is a chronic autoimmune disease of the central nervous system caused by various factors. The MS pathological process involves breakdown of the blood brain barrier, axonal degeneration, extensive demyelination, oligodendrocyte loss, reactive gliosis, and multifocal inflammation. Specifically, chronic inflammation, accompanied by the activation of microglia and the continuous participation of lymphocytes, is a representative feature of pathophysiology (111, 112). Although the exact cause is unclear, autoreactive T cells play a crucial role in inducing tissue damage in MS (113). Experimental autoimmune encephalomyelitis (EAE) mouse model is usually used to study the underlying cellular and molecular mechanisms of MS.

The disease process of MS is diverse. However, there were no reliable circulating biomarkers for predicting disease outcome of MS. Previous studies have demonstrated that TIGIT^-/-^ mice were more susceptible to EAE (7). Loss of TIGIT in a susceptible background result in hyperproliferative T cell responses and lead to the spontaneous development of EAE in 2D2×TIGIT^-/-^ mice (7). TIGIT was thought to reduce T cell responses indirectly by inducing tolerogenic DCs. Joller et al. generated an agonistic anti-TIGIT mAbs and demonstrated that activation of TIGIT could directly inhibit T cell responses independent of APCs by attenuating TCR-driven signals (7). Therefore, TIGIT might be involved in the maintenance of peripheral tolerance and this pathway plays a pivotal role in limiting autoimmune responses.

Burton et al. developed a dose escalation strategy for self-antigen-specific tolerance induction to modulate the phenotype and function of CD4^+^ T cells at each consecutive stage of escalating dose immunotherapy (EDI). They used MHC binding MBP peptide (MBP Ac1-9[4Y]) and Tg4 TCR transgenic model of EAE to investigate antigen-specific CD4^+^ T cell responses. The results demonstrated that EDI could effectively induce tolerance, minimize the activation and proliferation of CD4^+^ T cells and prevent excessive systemic cytokine release (114). IL-10 is well known for its role in limiting immune pathology and is associated with immunotherapy of autoimmune diseases (115). The use of EDI could promote the secretion of IL-10 and enhance the percentage of TIGIT^+^ cells. Moreover, a positive correlation between the production of IL-10 and the expression of PD-1, TIGIT, LAG-3, and TIM-3 was found in this study (114). This study demonstrated that TIGIT expressing T cells are strongly associated with the inhibition of EAE and are induced through antigen-specific immunotherapy (114). Another report showed that activated TIGIT signaling pathway reduces Th1 differentiation included 9 MS patients and 7 healthy controls, indicating that TIGIT stimulation regulates the production of IFN-γ and restores the inhibitory function of Th1 Tregs from patients with MS (18).

Based on the important roles of co-inhibitory receptors in regulating the termination of immune responses and autoimmunity, Lavon et al. initially investigated the potential of co-inhibitory molecules as predictive biomarkers and prognostic indicators of MS (116). In a sample size of 57 MS patients and 19 HCs, lower levels of TIGIT and LAG-3 were found on CD4^+^ T in patients with MS compared to that of the HCs. The expression levels of LAG-3 and TIM-3 correlated with MS outcome measures (116). The data indicate that the blood levels of co-inhibitory receptors might be efficient biomarkers of disease prognosis in MS and a more complete personalized therapy schedule could be specified by using these markers. Thus, these studies suggest TIGIT-expressing T cells are involved in pathology of MS and could be targeted for immunomodulatory therapies.

### 3.6 Psoriasis

Psoriasis is a chronic inflammatory disorder of the skin characterized by epidermal inflammation, angiogenesis, excessive growth and aberrant differentiation of keratinocytes. Previous studies have shown that T cells, DCs and inflammatory cytokines are involved in the occurrence and development of this disease (117, 118). TIGIT expression levels on CD4^+^ T cells of psoriasis vulgaris (PV) patients are significantly reduced and it is negatively correlated with psoriasis area and severity index. As we all know, TIGIT plays a vital role in inhibiting T cell proliferation and changing cytokine balance. Thus the low expression of TIGIT in PV patients could affect the expression levels of inflammatory factors such as IL-10, IL-17A and IFN-γ. In an experiment on CD4^+^ T cells in the peripheral blood of PV patients, activation of TIGIT-related signal pathways by recombinant human CD155-Fc protein can significantly inhibit the proliferation, resulting in a decrease in IFN-γ and IL-17A levels, and an increase in IL-10 levels (119). Strangely, after blocking the TIGIT signaling pathway with functional anti-human TIGIT antibody, the secretion of IFN-γ and IL-17A increased, but there was no significant change in IL-10 or cell proliferation. Therefore, the differential expression of TIGIT affects the balance of psoriasis cytokines and the proliferation of CD4^+^ T cells, this also suggests that the down-regulation of TIGIT on CD4^+^ T cells may promote the occurrence of psoriasis and it also provides a new method for the treatment of psoriasis, activating the TIGIT signaling pathway (119).

### 3.7 Primary Sjögren’s Syndrome

Primary Sjögren’s syndrome (pSS) is a chronic autoimmune disorder characterized by the infiltration of lymphocytes in the exocrine glands, mainly salivary glands and lacrimal glands, causing dryness in the eyes and mouth (120). In pSS, the typical feature of exocrine gland lesions is the formation of ectopic germinal center-like structures (121). Although the exact mechanisms underlying the disease remain unclear, numerous evidences showed that the hyperactivity of T cells might play a critical role in the pathogenesis of pSS (122, 123). Therefore, it is important to understand the regulatory mechanism that affects T cell activation.

CD226 promotes the pro-inflammatory capacity of effector T cells but inhibits the suppression of immune response whereas TIGIT possess the opposite function. Therefore, the balance between CD226 and TIGIT is important to maintain immune homeostasis (7, 124). A recent study has shown that the frequencies of CD226/TIGIT expressing CD4^+^ and CD8^+^ T cells were significantly higher in patients with pSS than in HCs and other rheumatic disease controls and they were associated with disease activity of pSS (14). Significant increase in the percentages of CD4^+^CD226^+^ and CD4^+^TIGIT^+^ T cells was observed in the active pSS compared to those in the inactive patients suggesting the specific role of these pathways in the pathogenesis of pSS (14). The proportion of CD4^+^TIGIT^+^ T cells is positively correlated with the erythrocyte sedimentation rate. Nevertheless, there was no such correlation in regard to the proportion of CD226/TIGIT in CD8^+^ T cells. CD4^+^TIGIT^+^ T cells showed enhanced activity than the CD4^+^TIGIT^-^ T cells in pSS patients (14). Therefore, the proportional alteration of CD226/TIGIT expressing CD4^+^ T cells could be a potential therapeutic target for pSS.

### 3.8 Aplastic Anemia

Aplastic anemia (AA) characterized by pancytopenia and bone marrow hypoplasia is also defined as a kind of autoimmune diseases. Although there are many factors inducing AA, T cell activation is considered to play an important role in the pathological of this process. Among different subtypes of T cells, most interest points to Th1 cells because they are expanded in patients with AA (125). Zhang et al. found that the percentage of TIGIT-positive CD4^+^ T cells was significantly reduced in AA patients (85%, 17/20) compared to that of healthy donors. Similarly, a decline in TIGIT-expressing CD4^+^ T cells in the spleen of AA mouse was observed. These findings suggest the decreased expression of TIGIT in CD4^+^ T cells might confer the pathogenesis of AA. T-bet was reported to be elevated in peripheral blood T cells from patients with AA (126). Th1 cells are recruited into the bone marrow to destroy hematopoietic stem cells by secreting cytokines (125). Overexpression of TIGIT by lentivector infection of human CD4^+^T cells, the cell proliferation, cytokines secretion (IL-12, IFN-γ, and TNF-α), and T-bet expression were significantly hindered whereas IL-10 production was enhanced. *In vivo* study also showed that TIGIT over-expressed CD4^+^ T cells adoption rescued the decrease red blood cell count and alleviated immune-mediated bone marrow failure of AA in mouse model (127). This study combined the clinical samples and *in vivo* animal experiment, which demonstrated that overexpression of TIGIT could inhibit the function of CD4^+^ T cells and alleviate AA in mouse models.

### 3.9 Autoimmune Uveitis

Autoimmune uveitis (AU), a group of diseases defined as intraocular inflammation, is a major cause of blindness in humans. The inflammation in uveitis is non-infectious in the majority of cases (128). Through the animal model of experimental autoimmune uveitis (EAU), we have a certain understanding of the mechanism of this disease. Th1 and Th17 subsets of CD4^+^ T cells contribute to the inflammation in EAU while Tregs suppress the autoreactive T-cells *via* secreting anti-inflammatory cytokines such as IL-10 and TGF-β. It was found that the frequency of Tregs is increased during the disease resolution of EAU (129, 130). However, the suppressive effects of Tregs was weaker in animals with recurrent EAU (129). A decrease of peripheral blood levels of Tregs was observed in patients with active uveitis and the levels were up-regulated during the process of remission (131, 132). Tregs expressing TIGIT were demonstrated to selectively inhibit Th1 and Th17 responses, but not Th2 (8).

The dysfunction of Tregs in the pathogenesis of AU was well described. It has been identified that different subsets of Tregs utilize distinct mechanisms of suppression to prevent autoimmune diseases (133, 134). A study involving 50 AU patients and 10 healthy subjects revealed that there were significantly higher frequencies of CD4^+^CD25^+^FoxP3^+^ Treg, TIGIT^+^ Treg, and T-bet^+^ Treg and Treg/Th1 ratio in the clinical remission subjects compared with active patients of uveitis (135). And higher serum levels of IL-10 and TGF-β were also associated with clinical remission. The increased expression of TIGIT is associated with hypomethylation and FOXP3 binding at the locus of TIGIT (28), and TIGIT^+^ Tregs contributed to the remission of autoimmune uveitis. The clinical criteria to classify uveitis has been formulated by standardization of Uveitis Nomenclature (SUN) working group. However, the specific immunological biomarkers are required to be defined. In this clinical study, the levels of TIGIT^+^ Tregs in peripheral blood were a sensitive biomarker (with a sensitivity of 92%) of clinical remission in sight-threatening non-infectious uveitis (135). Therefore, the up-regulation of immunoregulatory phenotype of Tregs associated with clinical remission of uveitis, which may assist with individualized therapy of in future. Muhammad et al. identified a novel subset of TIGIT^+^FoxP3^+^ Tregs that possesses a suppressive function (15). The number of TIGIT^+^FoxP3^+^ Tregs was significantly decreased in PBMCs from patients with uveitis (15). Results further showed that TIGIT^+^FoxP3^+^ Tregs are induced by stimulation of adenosine 2A receptor (A2Ar) in healthy volunteers, but not in patients with uveitis (15). In post-EAU mice with deficiency of A2Ar had fewer TIGIT^+^FoxP3^+^ Tregs in spleen compared with post-EAU wild type mice. And the animal results showed that a defect in the induction of TIGIT^+^ Tregs through A2Ar may contribute to chronic uveitis (15).

### 3.10 Autoimmune Thyroid Disease

Autoimmune thyroid disease (AITD) is one of the most common representatives of autoimmune diseases. Impaired numbers and dysfunctional of Tregs have been observed in patients with AITD (136). The suppressive phenotype and function of Tregs were investigated in an experimental autoimmune thyroiditis (EAT) mouse model. OX40L-JAG1 treatment significantly increased the frequencies of CTLA4^+^Foxp3^+^ and TIGIT^+^Foxp3^+^ Tregs in the spleen and lymph nodes and alleviated EAT. Enhanced Treg suppressive effects were observed in OX40L-JAG1 expanded subjects compared with the cells in control groups (137). These results suggest that TIGIT^+^ Tregs play an important role in AITD, but future work is required to elucidate the diagnostic and therapeutic potential of TIGIT^+^ Tregs in AITD.

### 3.11 Dermatomyositis

Dermatomyositis (DM) is an autoimmune inflammatory disease, which is characterized by myositis and skin manifestations. It is a heterogeneous disorder that can also affect other organ systems such as the cardiovascular, pulmonary, and gastrointestinal systems. Predominant infiltration of T cells was observed in muscle biopsies suggesting that T cells dysregulation plays a crucial role in DM pathogenesis but the exact mechanisms remain unclear (138, 139). The expression of immune checkpoint receptors has been shown to play a critical role in proper contraction of effector T cell responses (4). In a recent study, an altered balance between co-stimulatory and co-inhibitory molecules on T cells might contribute to pathogenesis of DM. A significantly elevated frequency of TIGIT^+^CD226^+^ CD4 T with enhanced effector function was observed in patients with DM (140). However, no significant difference in the expression of TIGIT/CD226 was found on CD8^+^ T cells between DM and HCs. The percentage of these cells was positively correlated with the disease activity of DM by Myositis Disease Activity Assessment (MYOACT) scores. Recombinant proteins CD112, CD155, and anti-CD226 Abs were used to examine the potential therapy for DM by intervening TIGIT^+^CD226^+^ CD4 T *in vitro*. Among them, anti-CD226 Abs remarkably suppressed the stimulatory function by inhibiting the production of TNF-α and IFN-γ by TIGIT^+^CD226^+^ CD4 T cells in both DM patients and HCs. However, treatment with CD112 and CD155 only slightly decreased TNF-α production with no significant differences (140). Therefore, these results provided insight into the therapeutic target of the TIGIT/CD226 axis by CD226 blockade in DM (**Table 1**).

**Table 1:** Expression profile and potential roles of TIGIT in autoimmune diseases.

Disease	Species	Cell type	Expression	Function	Ref.
RA	Human	CD4^+^ T	TIGIT is lower in active RA than inactive RA	Negative correlation between TIGIT expression and RA disease activity	(56)
RA	Human	CD4^+^ T	TIGIT was increased in RA SFMCs compared to both RA and HC PBMCs.	The high expression of IR on T cells in microenvironment of RA joint favoring T cell exhaustion	(61)
RA	Human	Treg	TIGIT is elevated in CD4^+^Foxp3^+^T cells in RA patients	It was not associated with disease activity of RA patients	(27)
RA	Mouse	P2rx7^-/-^Tfh	The expression of TIGIT is increased on P2rx7^-/-^ Tfh cells	P2RX7 promotes the death of Tfh cells by inhibiting TIGIT mediated anti-apoptotic effect	(10)
RA/SLE	Human	NK cell	Levels of TIGIT on NK cells are significantly lower in patients with RA/SLE than HC	TIGIT expression is inversely correlated with the IFN-γ-producing capability of NK cells	(71)
SLE	Human	Neutrophil	There is no significant difference in TIGIT^+^ neutrophils frequency between SLE and HC	No evaluated	(84)
SLE	Mouse	Tfr cell	The expression levels of TIGIT are highest on Tfr cells within Treg population	Tfr cells with high expressing of TIGIT effectively repress the production of anti-dsDNA IgA in pristane-induced lupus model	(80)
SLE	Human	CD4^+^T	TIGIT expression was significantly elevated in SLE patients	Expression levels of TIGIT are highly correlated with activity of SLE	(79)
SLE	Human	CD3^+^CD4^+^ T	The frequency of TIGIT-expressing was elevated in patients with SLE	The frequency of TIGIT-expressing CD3^+^CD4^+^ T cells was associated with the disease activity in SLE	(32)
SLE	Human	CD4^+^ T	A decreased frequency of TIGIT^+^CD4^+^ T cells was found in SLE with renal manifestations	It might support the dampened homeostasis maintenance	(11)
SLE	Human	NK cell	TIGIT expression was significantly decreased on NK cells	It was negatively correlated with the SLEDAI	(81)
IBD	Human	CD38^+^ Te	TIGIT frequency in CD38^+^ Te cells are reduced in a subgroup of pediatric IBD patients	TIGIT expression identifies circulating CD38^+^ Te cells with immunoregulatory properties	(88)
IBD	Human	CD4^+^T, CD8^+^ T	Surface levels of TIGIT was lower on both mucosal CD4^+^ and CD8^+^ T cells of IBD patients than that of the control samples	It regulates the activation and function of mucosal T cells and might reflect the disease activity of IBD	(12)
IBD	Mouse	Treg	Lower frequency of TIGIT^+^ Tregs in spleen and other tissues in colitis mice than wild type	TIGIT could reflect the activation status of Treg cells	(92)
IBD	Human	Treg	The percentage of CD226^+^TIGIT^+^FoxP3^+^ Tregs was increased in active UC	TIGIT might be a clinical biomarker of disease activity in patients with UC	(93)
IBD	Human	NK	An increased expression of TIGIT was observed on NK cells in UC patients	The activity of NK cells in UC patients can be modulated by TIGIT	(94)
T1D	Human	Treg	Induced human Tregs in humanized transgenic mice show increased expression of TIGIT	It could contribute to the prevention of islet autoimmunity at risk of T1D	(109)
T1D	Mouse	CD8^+^ T	A sphingosine-1 receptor agonist, CYM-5442, enhanced the gene expression of TIGIT	It limits the ability of autoreactive T cells that enter the islets and kill β cells	(100)
T1D	Human	CD8^+^ T	CD8^+^ T cells in the patients who response best to teplizumab treatment expressed high level of TIGIT	It help to sustain T cells exhaustion in the therapy for T1D	(104)
T1D	Human	CD8^+^T	The percentage of partially exhausted KLRG1^+^TIGIT^+^ CD8^+^ T cells increased after teplizumab treatment	Induction of TIGIT^+^CD8 T cells may be a favorable indicator for clinical outcome in T1D	(105)
T1D	Mouse	Treg	Expression of TIGIT was higher in TCR high-affinity Tregs	It is involved in the suppressive mechanisms to control tissue specific autoimmune responses	(110)
T1D	Human	NK	Elevated expression of CD226/TIGIT ratio was observed in NK cells from T1D patients compared to controls	It alters the balance between costimulatory and coinhibitory receptors in NK cells	(13)
MS	Mouse	CD4^+^ T CD8^+^ T	TIGIT expression was enhanced in activated T cells	Loss of TIGIT accelerates development of autoimmunity	(7)
MS	Mouse	CD4^+^ T	Percentage of TIGIT^+^ cells was increased during escalating dose immunotherapy	It correlates with IL-10 production in CD4^+^ T cells	(114)
MS	Human	CD4^+^ T	The expression of TIGIT was significantly decreased in MS patients	As potential prognostic indicator in MS	(116)
Psoriasis	Human	CD4^+^ T	Frequency of TIGIT^+^CD4^+^ T cells was lower in patients with psoriasis vs. HCs	It is negatively correlated with psoriasis severity index	(119)
pSS	Human	CD4^+^ T CD8^+^ T	The frequency of CD226/TIGIT on T cells was elevated in patients with pSS	It is associated with disease activity of pSS and could be a potential novel therapeutic target	(14)
AA	Human	CD4^+^ T	TIGIT-positive CD4^+^ T cells was reduced in AA patients	It might confer the pathogenesis of AA	(127)
AU	Human	Treg	The frequency of TIGIT^+^ Treg was significantly higher in remission patients vs. patients with active uveitic disease	It is a sensitive biomarker of clinical remission in non-infectious uveitis	(135)
AU	Mouse/ Human	Treg	Numbers of TIGIT^+^FoxP3^+^ Tregs were reduced in post-EAU mice and uveitis patients	The emergence of TIGIT^+^ Tregs induced by A2Ar contributes to resolution of uveitis	(15)
AITD	Mouse	Treg	Administration of OX40L-JAG1 expanded TIGIT^+^FoxP3^+^ Tregs in the spleen and LNs in EAT mice	They enhance the suppressive functions of Tregs and contribute to the amelioration of EAT mouse model	(137)
DM	Human	CD4^+^ T	The frequency of TIGIT^+^CD226^+^CD4 T cells was significantly elevated in patients with DM compared with HCs	It is positively correlated with DM disease activity	(140)

RA, rheumatoid arthritis; TIGIT, T cell immunoglobulin and ITIM domain; IR, inhibitory receptor; SFMC, synovial fluid mononuclear cell; HC, healthy control; PBMC, peripheral blood mononuclear cell; Treg, regulatory T cell; Tfh, helper T cell; SLE, systemic lupus erythematosus; Tfr, follicular regulatory T; SLEDAI, SLE disease activity index; IBD, Inflammatory bowel disease; Te, effector T cells; UC, ulcerative colitis; T1D, type 1 diabetes; TCR, T cell receptor; MS, multiple sclerosis; pSS, primary Sjögren’s syndrome; AA, aplastic anemia; AU, experimental autoimmune uveitis; EAU, experimental autoimmune uveitis; AITD, autoimmune thyroid disease; EAT, experimental autoimmune thyroiditis; LN, lymph nodes; DM, dermatomyositis.

### 3.12 Other Diseases

Currently, TIGIT is also playing a role in the area of transplantation. Hyporeactivity of T cells to donor antigens contributes to reduced risk of acute rejection in the years following kidney transplantation; However, the underlying mechanisms of donor-specific hyporesponsiveness (DSH) are largely unknown. Recent study about detection of exhaustion markers expressing circulating donor-reactive T cells with kidney transplant recipients by multi-parameterflow cytometry showed that the donor-reactive CD4^+^T cells highly expressing TIGIT are significant decrease long after kidney transplantation, suggesting it could be a marker of hypofunctional or exhausted T cells (141). Moreover, previous study has also indicated that the circulating TIGIT^+^Tregs are increased by donor-derived human regulatory macrophages (Mregs) to living-donor kidney transplant, which promotes allograft acceptance through rapid induction (142). Another study to identify the immune-related genes in kidney papillary cell carcinoma (pRCC) patients found that B cells had a higher percentage than other immune cells and TIGIT had a higher expression in the high-risk score group (143).

Interestingly, there is also high expression of TIGIT in allogeneic hematopoietic stem cell transplantation and CD8^+^ T cells from acute myeloid leukemia (AML) patients, where it may mitigate the severity of graft-versus-host disease (GVHD) and is associates with primary refractory disease and leukemia relapse post transplantation (144–146). In a recent study, the expression of TIGIT is different in reconstituted T cells in ABO- versus HLA-incompatible kidney transplant recipients, T cells reconstitution of patients with anti-human leukocyte antigen (HLA) an-tibodies have an increased expression of TIGIT, which is not associated with decreased cytokine production; Conversely, TIGIT expression was negatively associated with cytokine production in CD4^+^ T cells (147). These results suggest that TIGIT could have an impact on the transplantation outcome.

## 4 Novel Therapeutic Approaches *via* TIGIT Enforcement

**Figure 3:**
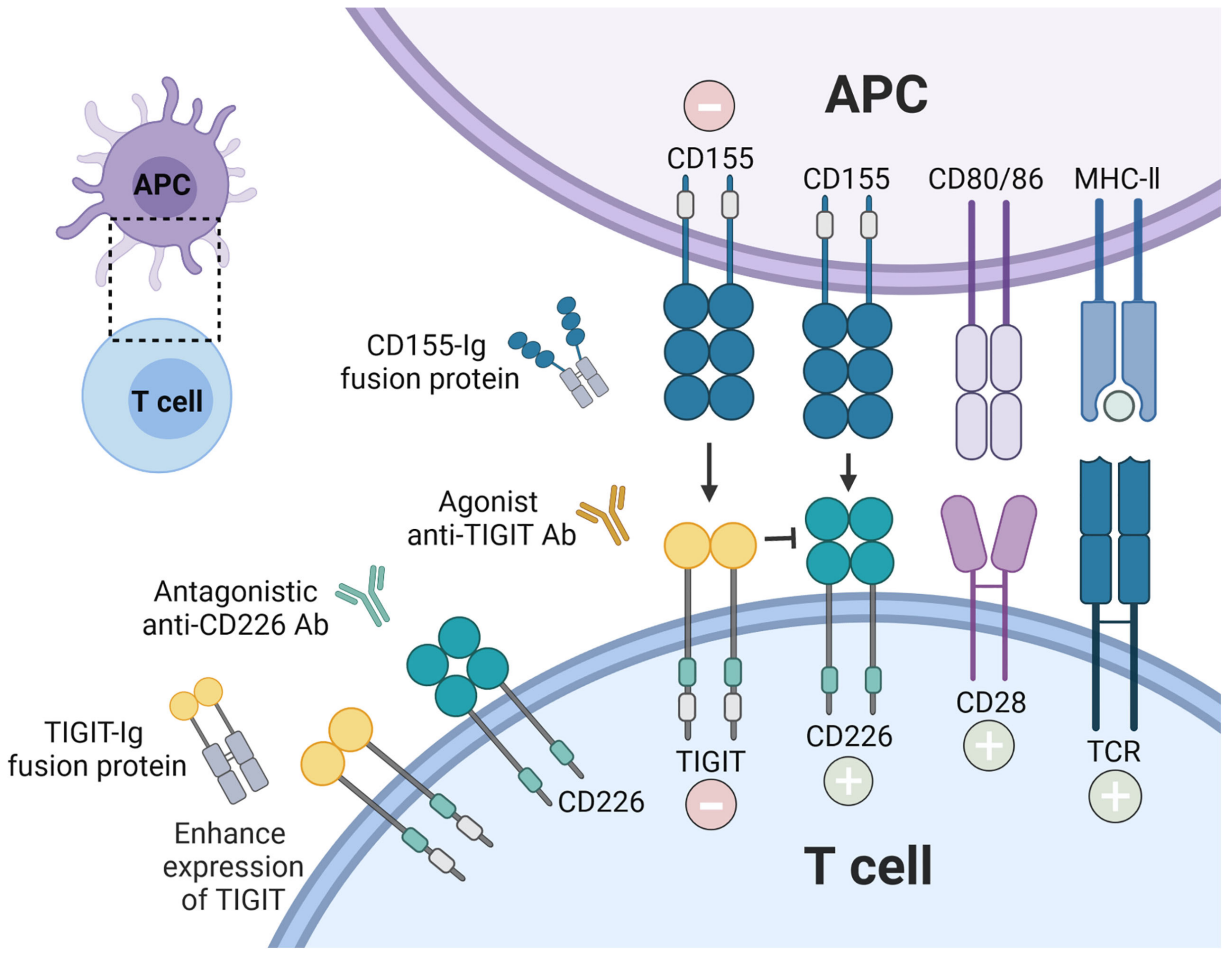
Therapeutic intervention by enforcing CD155-TIGIT axis. The current strategies are being investigated to block autoreactive T cell responses *via* TIGIT signaling pathway: recombinant TIGIT-Ig fusion protein; TIGIT overexpression; agonist anti-TIGIT Ab, and recombinant CD155. In addition, antagonistic anti-CD226 Ab could also inhibit the stimulatory function of T cells.

The above observations suggest that the phenotype and function of TIGIT expressing cells were changed in several autoimmune diseases. As a co-inhibitory receptor, TIGIT is like a biological brake of the immune responses. Previous studies in mouse model found that loss of TIGIT or blocking TIGIT signaling pathway led to the hyperproliferative T cells responses, aggravation of the inflammation, and promoted susceptibility to autoimmune diseases (7, 79). Increasing data for the roles of TIGIT in autoimmunity suggest that enforcement of TIGIT signaling and its downstream consequences might prevent or treat autoimmune diseases (**Figure 3** **)**. Different strategies such as TIGIT overexpression, agonistic anti-TIGIT mAb, recombinant CD155 protein, and TIGIT-Ig fusion protein have been used in mice models for treatment of autoimmune diseases (**Table 2**).

**Table 2:** TIGIT reinforcement studies aimed at treat autoimmune diseases.

Reinforcement method	Disease	Species	Disease setting	Ref.
TIGIT-Ig fusion protein	CIA EAE	Mouse	Administration of TIGIT Vstm3 Fc-fusion protein significantly inhibited the severity of CIA and EAE	(29)
TIGIT-Ig fusion protein	SLE	Mouse	Administration of TIGIT-Ig reduced autoantibodies production and prolonged survival of SLE mice	(16)
Lentivector infection to upregulate TIGIT	CIA	Mouse	TIGIT overexpression ameliorated the severity of RA and inhibited the production of anti-collagen II antibodies	(56)
TIGIT overexpression by plasmid transfection	AA	Mouse	TIGIT overexpression on CD4^+^T cells alleviated disease of AA in mouse models	(127)
Agonistic anti-TIGIT Ab	MS	Human	Agonist of TIGIT showed inhibitory effects on CD4^+^ T cells in MS patients	(148)
Agonistic anti-TIGIT Ab	EAE	Mouse	Treatment with the agonistic anti-TIGIT Ab reduced the disease severity in EAE mouse model	(17)
Recombinant Fc-CD155	SLE	Mouse	Activation of TIGIT by recombinant CD155 protein repaired the activities of CD4^+^ T cells and delayed the development of SLE	(79)
Recombinant Fc-CD155	MS	Human	Activation of TIGIT signaling by Fc-CD155 represses IFN-γ production and restores the functional stability of Tregs in MS	(18)

TIGIT, T cell immunoglobulin and ITIM domain; CIA, collagen-induced arthritis; EAE, experimental autoimmune encephalomyelitis; SLE, systemic lupus erythematosus; RA, rheumatoid arthritis; AA, aplastic anemia; MS, multiple sclerosis; EAE, experimental autoimmune encephalomyelitis.

### 4.1 Recombinant TIGIT-Ig Fusion Protein

Recombinant TIGIT-Ig fusion protein has been shown to attenuate T cell responses both *in vitro* and *in vivo*. The therapeutic function of sTIGIT was tested in different autoimmune disease models including CIA and in EAE (29). In CIA model, administration of the murine TIGIT tetramer or the Fc-fusion protein significantly alleviated the disease severity in treated mice, which is correlated with reduction of IL-6 production (29). For the effects of sTIGIT in CIA, mice treated with sTIGIT had significantly fewer CD4^+^ T cells expressing pro-inflammatory cytokines IL-17A and TNF-α in draining lymph nodes (DLN) and spleen, this data suggests TIGIT alleviates disease process through a general suppressing of CD4^+^ T cell responses, which most likely by interfering with CD226-mediated costimulation (29). Although both the tetrameric and dimeric fusion proteins possessed therapeutic effects, the tetramer showed better efficacy. In contrast, blocking TIGIT resulted in more rapid disease onset (29). It was reported that TIGIT deficient mice developed more severe EAE compared to wild-type mice. The effect of sTIGIT was also tested in MOG induced EAE model. As in CIA model, sTIGIT tetramer significantly attenuated disease symptoms while treatment with blocking anti-TIGIT mAb exacerbated disease development (29).

Liu et al. also generated recombinant TIGIT-Ig fusion protein and proved its therapeutic potential *in vivo*. In lupus-prone (NZB/NZW F1) mice model that was treated with TIGIT-Ig fusion protein, the onset of SLE was delayed, proteinuria and autoantibodies production were reduced, inflammatory response was inhibited and survival was prolonged compared to those of the controls (16). This fusion protein also shows a unique advantage, which can weaken the ability to cause host immune response, which is also the biggest difference from traditional biological agents. Therefore, TIGIT-Ig may become another effective way to treat autoimmune diseases (16). This data suggest that administration of TIGIT-Ig may be a promising prevention and treatment for SLE patients.

### 4.2 TIGIT Overexpression

In a RA mouse model induced by type-II collagen, TIGIT expression was upregulated *in vivo* by lentivector infection. Results showed that TIGIT overexpression effectively decreased the amount of anti-collagen II antibodies and alleviated the disease severity, which is closely related to the repaired function of CD4^+^ T cells (56). On the other hand, blockade or genetic ablation of TIGIT enhanced CD4^+^ T cells priming and exacerbated the disease severity of RA (56). The above evidence indicates the prospect of TIGIT in the clinical treatment of RA patients. TIGIT overexpression on CD4^+^ T cells by plasmid transfection significantly increased the counts of RBC, reduced the plasma levels of TNF-α and INF-γ, and up-regulated the expression of CD34, SCF, and GM-CSF in bone marrow mononuclear cells (BMMNCs) compared to the wild-type CD4^+^ T cell-induced AA mice, suggesting the roles of TIGIT overexpression in protect the bone marrow failure of AA (127). As expected, administration of TIGIT-overexpressed CD4^+^ T cells prolonged the median survival of AA mice and some mice among them even being fully rescued (127). Thence, overexpression of TIGIT mainly inhibited CD4^+^ T cells activation and cytokine secretion and played a positive role in RA and AA mouse models (55, 126). The above observations indicate that TIGIT expression enhancement could provide a novel potential strategy to ameliorate autoimmune diseases.

### 4.3 Agonistic Anti-TIGIT Ab

The roles of a panel of monoclonal anti-TIGIT Abs were evaluated in EAE mice model. Administration of the agonistic anti-TIGIT Ab is capable of regulating T cell responses *in vivo* and ameliorating the disease severity in EAE (17). These results demonstrate that treatment with agonistic anti-TIGIT Abs could dampen autoimmune T cell responses *in vivo* and the reduction of T cell expansion and proinflammatory cytokines leads to amelioration of MS (17). Furthermore, TIGIT is expressed at normal levels in patients with MS and the inhibitory effects of TIGIT signaling pathway on CD4^+^ T cells from MS patients are functional, suggesting the potential use of this agonistic Ab in clinical for MS (148). An agonistic Ab to TIGIT could directly inhibit CD4^+^ T cells proliferation in a T cell-intrinsic manner, with a reduce in T-bet, IRF4, GATA3 expression and a decrease in cytokines production, especially IFN-γ (148). While knockdown of TIGIT by shRNA increased the mRNA and protein levels of T-bet and IFN-γ on ex vivo CD4^+^ T cells which could be overcome by CD226 blocking. That may be explained by the mechanism that TIGIT competes with CD226 for CD155 ligand to exert its immunosuppressive function. These results indicate that TIGIT could not only directly T cell functions but also T cells by competing with CD226 (148). The Abs presented above can serve as potential tools for evaluate TIGIT function in autoimmune diseases and provide novel therapeutic strategies by modulating TIGIT pathway. In addition, anti-CD226 Ab significantly decreased the stimulatory function of TIGIT^+^CD226^+^ T cells from DM patients (140). Therefore, targeting TIGIT/CD226 axis might be a more efficient manipulation of T cells in autoimmune diseases.

### 4.4 Recombinant CD155

*In vivo* administration of recombinant CD155 protein resulted in a delayed development of SLE in MRL/lpr mice by impairing the activity of CD4^+^ T cells, including the expression of CD25, and CD69, production of IFN-γ, and proliferation, this data shows that activation of the TIGIT pathway can down‐regulate the activities of CD4^+^ T cells which reveals a new method to treat SLE (79). The frequency of Th1 Tregs which are characterized by impaired suppressor activity was increased in autoimmune diseases. IL-12 treatment was previously reported to recapitulate the features of MS-associated Th1 Tregs. TIGIT stimulation with Fc-CD155 reduced IFN-γ production and T-bet expression induced by IL-12 and restored the suppressor defect in Tregs from patients with MS (18). Stimulated TIGIT by Fc-CD155 functionally controls the Akt pathway *via* SHIP1 and leaded to loss of IFN-γ secretion from MS Tregs (18). While functional inhibition of FoxO1 or SHIP-1 abolished the protective effects of TIGIT. These data indicate the important role of TIGIT stimulation in correcting defects in autoimmune Tregs and could be targeted for therapy in human autoimmune disorders. Although extensive and in-depth studies are needed to test the therapeutic benefits of targeting co-inhibitory pathways, current understanding indicates that enforcement of TIGIT axis may offer a new therapeutic approach for the treatment of autoimmune diseases.

## 5 Conclusion

This work is focused on the characteristics of TIGIT and its roles in the regulation of autoimmune responses with the aim to provide novel therapeutic strategies in the treatment of autoimmune diseases. The link between TIGIT^+^ T/NK cells and autoimmunity has been documented in the literature. However, the effects of TIGIT on B cells were rarely studied. Regulatory B cells (Bregs) were shown to mitigate autoimmune diseases *via* immunosuppressive cytokines and immunoregulatory pathways including TIGIT (149). TIGIT expressed on B cells was demonstrated to control immune response through manipulating T cells and dampening pro-inflammatory effects of DC. The lack of TIGIT^+^ memory B cells was correlated with increased production of donor-specific antibody decreased response of Treg in liver allograft and renal patients (150). Likewise, TIGIT expression on B cells dependent on Tim-1 signaling are imperative in maintaining CNS-specific tolerance (151). These results suggested that TIGIT expressed on B cells is prone to control immune response thereby alleviating severity of autoimmunity. How to use the population of TIGIT^+^ cells as biomarker is very complicated owing to the heterogeneity of autoimmune diseases, disease status, individual differences of patients, etc. Immune checkpoint blockade is showing remarkable efficacy in cancers while it is accompanied by autoimmune disease-like side effects. It also proves the important roles of these inhibitory receptor associated signaling pathways in autoimmune diseases. Studies in several murine models have clearly demonstrated that enforcement of TIGIT signal pathway could be used for the treatment of these diseases. However, more evidence is need to support the application of TIGIT enforcement in clinical trial for autoimmune diseases. The roles of TIGIT in immune modulation holds promise for the development of efficient therapeutic strategies to treat autoimmunity. More extensive studies are required to better understand the exact functions and mechanisms of this inhibitory receptor in different autoimmune disorders. An in-depth exploration of the complex interaction between co-stimulatory and co-inhibitory pathways, for example TIGIT/CD226 combination, may be particularly important to allow optimal therapeutic selection and to ensure maximal clinical efficacy.

## Author Contributions

CY, SG, and CH drafted the manuscript. WW and CH conceived and designed the review outline. All authors contributed to the writing and critical review of the manuscript. All authors read and approved the final manuscript.

## Funding

This work was supported by National Natural Science Foundation of China [81901660, 81802963]; China Scholarship Council [201808330646]; Zhejiang Medical Science Foundation [2018KY531] and Lin He’s New Medicine and Clinical Translation Academician Workstation Research Fund [18331215].

## Conflict of Interest

The authors declare that the research was conducted in the absence of any commercial or financial relationships that could be construed as a potential conflict of interest.

## Publisher’s Note

All claims expressed in this article are solely those of the authors and do not necessarily represent those of their affiliated organizations, or those of the publisher, the editors and the reviewers. Any product that may be evaluated in this article, or claim that may be made by its manufacturer, is not guaranteed or endorsed by the publisher.
